# P02.123. The anti-diabetic and cholesterol-lowering effects of common and cassia cinnamon (Cinnamomum verum and C. aromaticum): a randomized controlled trial

**DOI:** 10.1186/1472-6882-12-S1-P179

**Published:** 2012-06-12

**Authors:** J Dugoua, D Perri, D Seely, J Ardilouze, R Ridout, K Bowers, T Einarson, G Koren

**Affiliations:** 1 University of Toronto, Toronto, Canada; 2 McMaster University, Hamilton, Canada; 3 Canadian College of Naturopathic Medicine, Toronto, Canada; 4 Centre Hospitalier Universitaire de Sherbrooke, Sherbrooke, Canada; 5 Toronto Western Hospital, Toronto, Canada; 6 University North Texas, Denton, USA

## Purpose

According to the World Health Organization (WHO), approximately 150 million people worldwide have type 2 diabetes. It is a growing health concern. Common and cassia cinnamon have been reported to have anti-diabetic and lipid-lowering effects. The objective was to determine if the combination of common and cassia cinnamon (Cinnamomum verum and C. aromaticum) reduces fasting blood glucose, insulin, glycosylated hemoglobin (HA1C), triglyceride, total cholesterol, HDL cholesterol and LDL cholesterol levels in people with type 2 diabetes.

## Methods

Fifty (50) type 2 diabetic participants were randomized to receive either 140 mg of Cinnamonforce twice daily or placebo over 13 weeks. Physical and laboratory measurements were taken at baseline, 2 weeks, 4 weeks, 8 weeks and at the end of the trial, 13 weeks.

## Results

There were no significant improvements in fasting glucose, insulin and lipid parameters between treatment and placebo groups. At endpoint, subjects in the treatment group were found to have a marginally non-significant higher fasting blood glucose level than subjects taking the placebo (p=0.085). There was a non-significant decrease in HA1c in the treatment group versus the placebo group (p=0.172). In secondary outcomes, significant differences in weight (p= 0.008), BMI (p= 0.001) and waist-to-hip ratio (p=0.020) were detected in the treatment versus placebo groups.

## Conclusion

The combination of common and cassia cinnamon do not impact fasting glucose, insulin and lipid measurements. Through power calculations, there may be an effect on HA1c, however, the sample size in this study was not sufficient to detect a trend, if any. Although secondary outcomes in this study, common and cassia cinnamon should be further investigated for weight loss.

